# Perillaldehyde Functions as a Potential Antifungal Agent by Triggering Metacaspase-Independent Apoptosis in Botrytis cinerea

**DOI:** 10.1128/spectrum.00526-23

**Published:** 2023-05-16

**Authors:** Guanbo Wang, Yadi Wang, Kunchun Wang, Haonan Zhao, Mengjie Liu, Wenxing Liang, Delong Li

**Affiliations:** a Shandong Engineering Research Center for Environment-Friendly Agricultural Pest Management, College of Plant Health and Medicine, Qingdao Agricultural University, Qingdao, China; b The Linzi Center for Agricultural and Rural Development, Zibo, China; Beijing Forestry University

**Keywords:** *Botrytis cinerea*, perillaldehyde, antifungal agent, ubiquitination, autophagy, metacaspase

## Abstract

Botrytis cinerea, the causal agent of gray mold, is an important plant pathogen causing preharvest and postharvest diseases. Due to the extensive use of commercial fungicides, fungicide-resistant strains have emerged. Natural compounds with antifungal properties are widely present in various kinds of organisms. Perillaldehyde (PA), derived from the plant species Perilla frutescens, is generally recognized as a potent antimicrobial substance and to be safe to humans and the environment. In this study, we demonstrated that PA could significantly inhibit the mycelial growth of B. cinerea and reduced its pathogenicity on tomato leaves. We also found that PA had a significant protective effect on tomato, grape, and strawberry. The antifungal mechanism of PA was investigated by measuring the reactive oxygen species (ROS) accumulation, the intracellular Ca^2+^ level, the mitochondrial membrane potential, DNA fragmentation, and phosphatidylserine exposure. Further analyses revealed that PA promoted protein ubiquitination and induced autophagic activities and then triggered protein degradation. When the two metacaspase genes, *BcMca1* and *BcMca2*, were knocked out from *B. cinerea*, all mutants did not exhibit reduced sensitivity to PA. These findings demonstrated that PA could induce metacaspase-independent apoptosis in *B. cinerea*. Based on our results, we proposed that PA could be used as an effective control agent for gray mold management.

**IMPORTANCE** Botrytis cinerea causes gray mold disease, is considered one of the most important dangerous pathogens worldwide, and leads to severe economic losses worldwide. Due to the lack of resistant varieties of *B. cinerea*, gray mold control has mainly relied on application of synthetic fungicides. However, long-term and extensive use of synthetic fungicides has increased fungicide resistance in *B. cinerea* and is harmful to humans and the environment. In this study, we found that perillaldehyde has a significant protective effect on tomato, grape, and strawberry. We further characterized the antifungal mechanism of PA on *B. cinerea*. Our results indicated that PA induced apoptosis that was independent of metacaspase function.

## INTRODUCTION

The necrotrophic fungus Botrytis cinerea Pers.: Fr. is one of the most important plant pathogens worldwide and has an exceptionally wide host range of cultivated plants (approximately 1,400 species) (1). This fungus can infect many fresh fruits and vegetables, causing severe economic losses, not only on field production but also on postharvest storage and preservation (2). Due to the lack of resistant varieties, gray mold control has mainly relied on application of synthetic fungicides. However, long-term and extensive use of synthetic fungicides has increased fungicide resistance in B. cinerea and is harmful to humans and the environment (3). Therefore, under the current circumstances, the development of alternatives to synthetic fungicides is highly demanded.

Use of plant extracts for plant pathogen control is considered an environmentally friendly alternative to the use of synthetic fungicides. Aromatic plant species such as Origanum vulgare and Eugenia caryophyllata have been recognized to possess antimicrobial properties. Carvacrol, a hydrophobic terpene component from O. vulgare, has been reported to be able to damage cell membranes, decrease intracellular ATP concentrations, and reduce the pathogenicity of the plant bacterial pathogen Dickeya zeae (4). Eugenol extracted from E. caryoplyllata could reduce the incidence and severity of peach soft rot caused by the fungal pathogen Rhizopus stolonifera (5). In tomato plants, eugenol has been found to induce H_2_O_2_ accumulation, increasing resistance to tomato yellow leaf curl virus (TYLCV) (6). Another natural antimicrobial substance, allicin (diallyl disulfide), has been reported to be able to inhibit plant pathogens. By changing the plant endogenous phytohormones, allicin can induce cucumber resistance to downy mildew caused by Pseudoperonospora cubensis. It is difficult for the pathogens to obtain allicin resistance and as a consequence, allicin has been used to control gray mold in table grape and strawberry (7, 8). In addition, allicin can affect cell division and promote root growth in tomato (9). Perillaldehyde (PA) is a natural monoterpenoid compound extracted from *Perilla frutescens*, which has been historically used as a popular leafy vegetable. A previous study reported that PA could induce Aspergillus flavus apoptosis through a metacaspase-mediated mitochondrial pathway (10).

Many drugs can induce cell apoptosis in yeast through reactive oxygen species (ROS) generation (11). Metacaspases are recognized as the main executors of stress-induced or age-induced apoptosis. In yeast, the metacaspase Yca1 stimulates programmed cell death in response to oxidative stress and clears insoluble protein aggregates against toxic drugs. Likewise, only a single metacaspase has been identified in Ustilago maydis, which is closely associated with insoluble intracellular protein aggregates and is involved in apoptosis-like programmed cell death (12). Two metacaspases were identified in Magnaporthe oryzae. Knockout of the two metacaspase genes resulted in delayed conidial germination, attenuated disease severity, and increased accumulation of insoluble aggregates. Furthermore, apoptosis in the double mutant was attenuated under oxidative stress conditions (13). Five metacaspases have been identified in the protist Trypanosoma brucei. Only three of the five contain a cysteine-histidine catalytic dyad with catalytic activity. However, these three metacaspases lack a role in programmed cell death. The metacaspase Mca1 lacks cysteine peptidase activity. Knockdown of Mca4 by RNA interference (RNAi) markedly reduced the virulence of T. brucei in mice. Therefore, Mca4 was regarded as a membrane-associated pseudopeptidase virulence factor (14–16).

It has been reported that PA has antifungal properties inhibiting the growth of A. flavus, Aspergillus niger, Aspergillus oryzae, and Alternaria alternata. In A. flavus, PA caused cell apoptosis and induced the secretion of cell wall-degrading enzymes. However, detailed insights into the mechanisms of the function of PA on *B. cinerea* are not clear. In this study, we conducted *in vitro* and *in vivo* assays to assess the effects of PA on *B. cinerea* and found that PA significantly inhibited the mycelial growth and pathogenicity of *B. cinerea*. PA was also found to be able to induce selective autophagy, protein degradation, and cell apoptosis, which further caused the loss of mitochondrial membrane potential and impairments of membrane integrity. The two metacaspase genes were deleted. In both the single-gene deletion and double-gene deletion strains, PA-induced apoptosis was inhibited. However, the growth and pathogenicity of the mutants had not become insensitive to PA, which indicated a metacaspase-independent nature of PA-induced apoptosis in *B. cinerea*. These results will help us to understand antimicrobial mechanisms of plant-derived substances and provide potential alternatives for better plant disease management.

## RESULTS

### PA inhibits mycelial growth and pathogenicity of *B. cinerea*.

**FIG 1:**
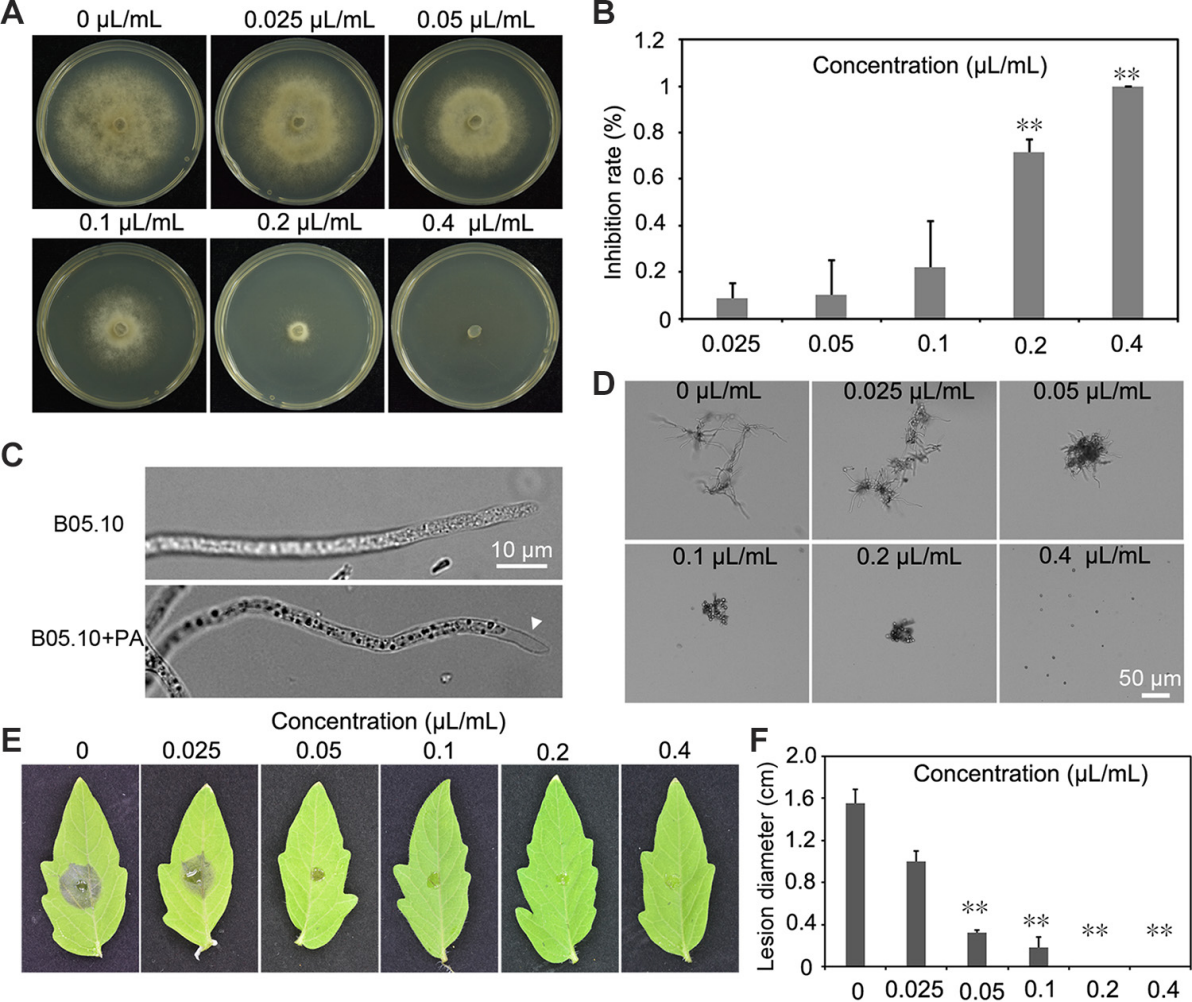
Inhibitory effects of PA on mycelial growth and the pathogenicity of Botrytis cinerea. (A) PA inhibits the mycelial expansion of *B. cinerea* after 4 days of growth on PDA plates supplemented with increasing concentrations of PA. (B) Statistical analysis of colony diameters. (C) Mycelial morphologies of *B. cinerea* grown in YEPD medium supplemented with PA. (D) *B. cinerea* conidia treated with PA under light microscopy after 12 h of incubation. (E) *In vivo* test of the inhibitory activity of PA against *B. cinerea* on detached tomato leaves. The leaves were treated with PA, inoculated with a *B. cinerea* conidial suspension, and photographed at 3 dpi. (F) Statistical analysis of lesion diameters. Asterisks represent statistically significant differences (*P* < 0.01). The experiments were performed in triplicate, and the data are shown as means ± standard deviations.

The antifungal activity of PA was investigated on potato dextrose agar (PDA) plates containing *B. cinerea* culture treated with different concentrations of PA. Based on the measurement after 3-day incubation, PA was found to be able to suppress the growth of colonies in a concentration-dependent manner. PA could completely inhibit the growth of strain B05.10 when the concentration reached 0.4 μL/mL (Fig. 1A and B). PA also showed antimicrobial activities against other pathogens, including Fusarium oxysporum, Colletotrichum fructicola, Neopestalotiopsis clavispora, Rhizoctonia solani, and Corynespora cassiicola, an oomycete species (Phytophthora capsici), and a bacterial species (Ralstonia solanacearum) (see Fig. S1A and B in the supplemental material). Plasmolysis was also observed on the hyphal tips of PA-treated *B. cinerea* (Fig. 1C). The rate of conidial germination and the length of germ tube were also observed. The results indicated that PA inhibited conidial germination and germ tube elongation in a concentration-dependent manner (Fig. 1D). To further investigate the inhibitory activity of PA against *B. cinerea*, an *in vivo* pathogenicity assay was conducted on detached tomato leaves. As measured at 3 days postinoculation (dpi), PA was found to be able to significantly impair the pathogenicity of *B. cinerea* with concentration increased. When the concentration of PA reached 0.1 μL/mL, almost no disease was found on tomato leaves (Fig. 1E and F).

**FIG 2:**
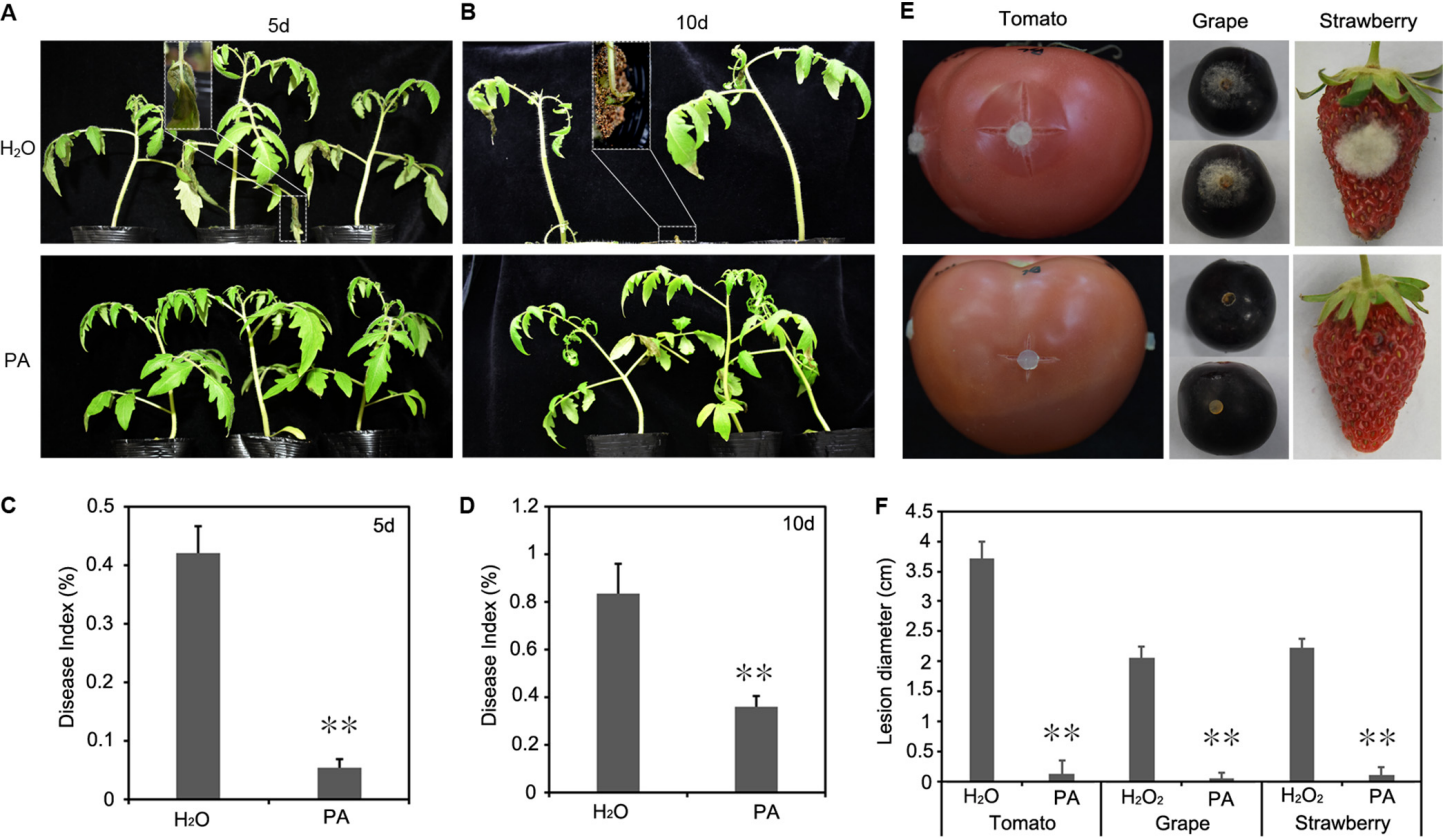
Inhibitory effects of PA against Botrytis cinerea on tomato plants and fruits of tomato, grapes, and strawberry. (A) *In vivo* test on tomato seedlings using the conidial suspension spraying approach. (B) Statistical analysis of the disease index data. (C) Lesion on harvested tomato, grapes, and strawberry 3 days after inoculation. (D) Lesion diameter was measured. Error bars represent the standard deviation with three replicates, and asterisks represent statistically significant differences (*P* < 0.01).

The inhibitory activity of PA against *B. cinerea* was further investigated on potted tomato. When 0.4 μL/mL of PA was applied on potted tomato, gray mold was significantly inhibited, with a disease index of 5.5 at 5 dpi (Fig. 2A and C). Conversely, the typical rotting symptoms of gray mold (disease index of 42.2) were observed on H_2_O-treated leaves. At 10 dpi, PA could reduce the incidence of gray mold, with a disease index of 36.1. In contrast, the disease index of H_2_O-treated leaves was 82.4 (Fig. 2B and D). The inhibitory activity of PA against *B. cinerea* was further confirmed on fruits of tomato, grape, and strawberry. Three days after inoculation with mycelial plugs or a conidial suspension, lesions were significantly smaller on PA-treated fruits than H_2_O-treated fruits (Fig. 2E and F). These results indicated that PA is a promising candidate botanical fungicide for gray mold management.

### PA induces apoptosis in *B. cinerea*.

**FIG 3:**
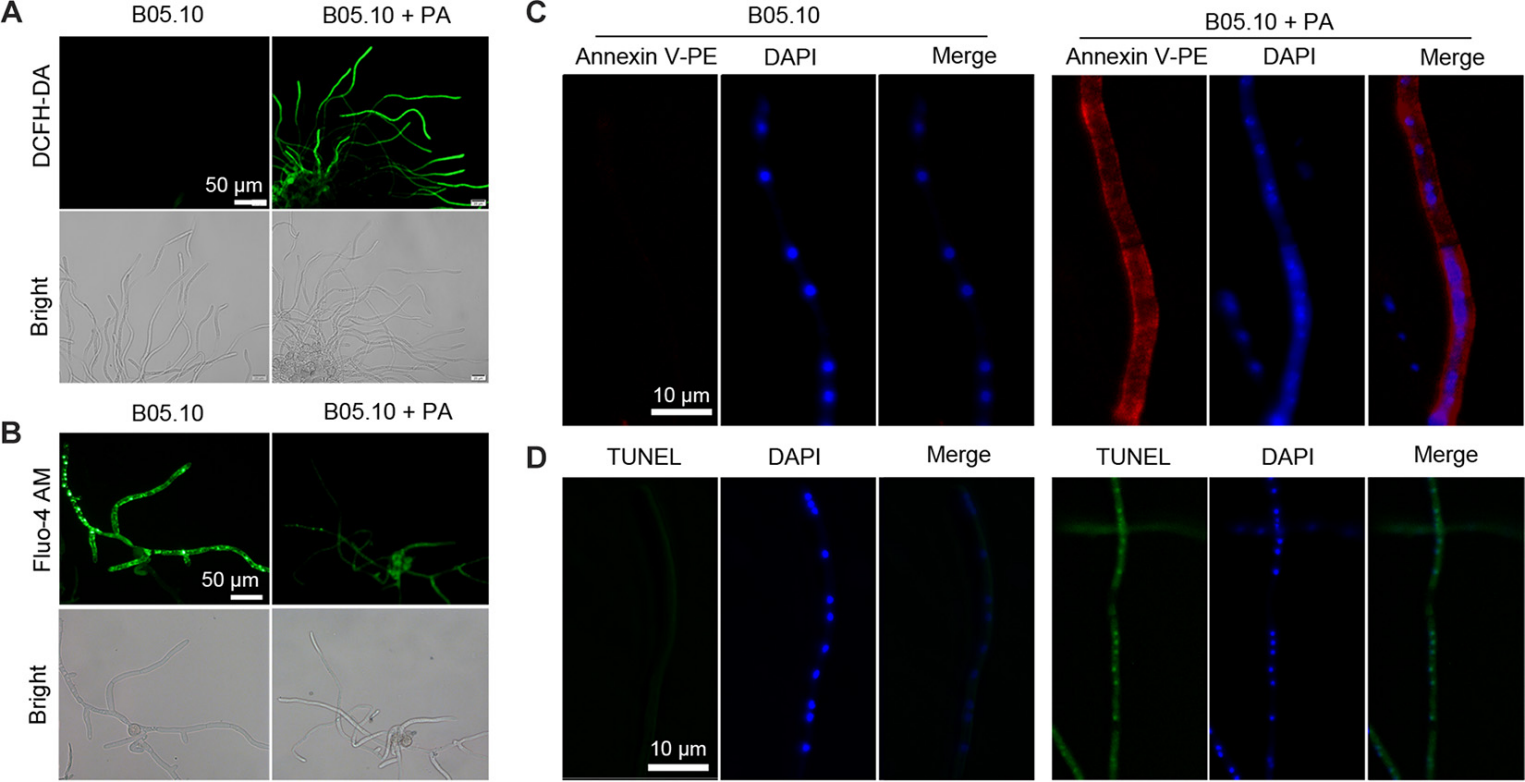
Induction of apoptosis by PA in Botrytis cinerea. (A) ROS accumulation was observed for *B. cinerea* hyphae at 4 h after PA treatment. Green fluorescence in the hyphae indicates ROS accumulation. (B) Elevation of intracellular Ca^2+^ in *B. cinerea* hyphae after PA treatment. (C) Hyphae at an early apoptosis stage were stained with annexin V-phycoerythrin (PE). Using DAPI staining, nuclear morphological change was examined. (D) DNA fragmentation induced by PA was measured with a TUNEL assay, and nuclear staining was done with DAPI.

To assess the effect of PA on apoptosis, intracellular ROS accumulation in the B05.10 strain after PA treatment was examined. The presence of 2,7-dichlorodihydrofluorescein diacetate (DCHF-DA) fluorescence indicated the cytosolic accumulation of ROS, and the fluorescence intensity represented the ROS production level in mycelia. The fluorescence intensity in the PA-treated mycelia was significantly higher than that in the H_2_O-treated mycelia (Fig. 3A). Calcium homeostasis is also important in apoptosis. Alteration in calcium levels was detected between the PA-treated mycelia and the H_2_O-treated mycelia. In the PA-treated mycelia, fluorescence mainly concentrated on/in the endoplasmic reticulum (ER) and was stronger than that of H_2_O-treated mycelia, in which the fluorescence was evenly distributed in the cytoplasm (Fig. 3B). These results indicated that calcium ions were released from the ER.

A previous study suggested that mycelia exposed to PA had an intracellular overload of reactive oxygen species (ROS), which could mediate apoptosis. Annexin V can be used to detect apoptosis by its ability to bind to phosphatidylserine, a marker of apoptosis when it is on the outer leaflet of the plasma membrane (17). Exposure of phosphatidylserine at the outer leaflet of the plasma membrane was found in the PA-treated mycelia (Fig. 3C); however, this was not observed in the H_2_O-treated mycelia. Another distinctive feature of late-stage apoptosis is DNA fragmentation. Therefore, DNA fragmentation in mycelia was assessed by terminal deoxynucleotidyl transferase dUTP nick end labeling (TUNEL) staining after treatment with PA. The PA-treated mycelia exhibited more green fluorescence in the nucleus, whereas the H_2_O-treated mycelia showed only slight fluorescence (Fig. 3D). These results indicated that PA plays an important role in apoptosis in *B. cinerea*.

**FIG 4:**
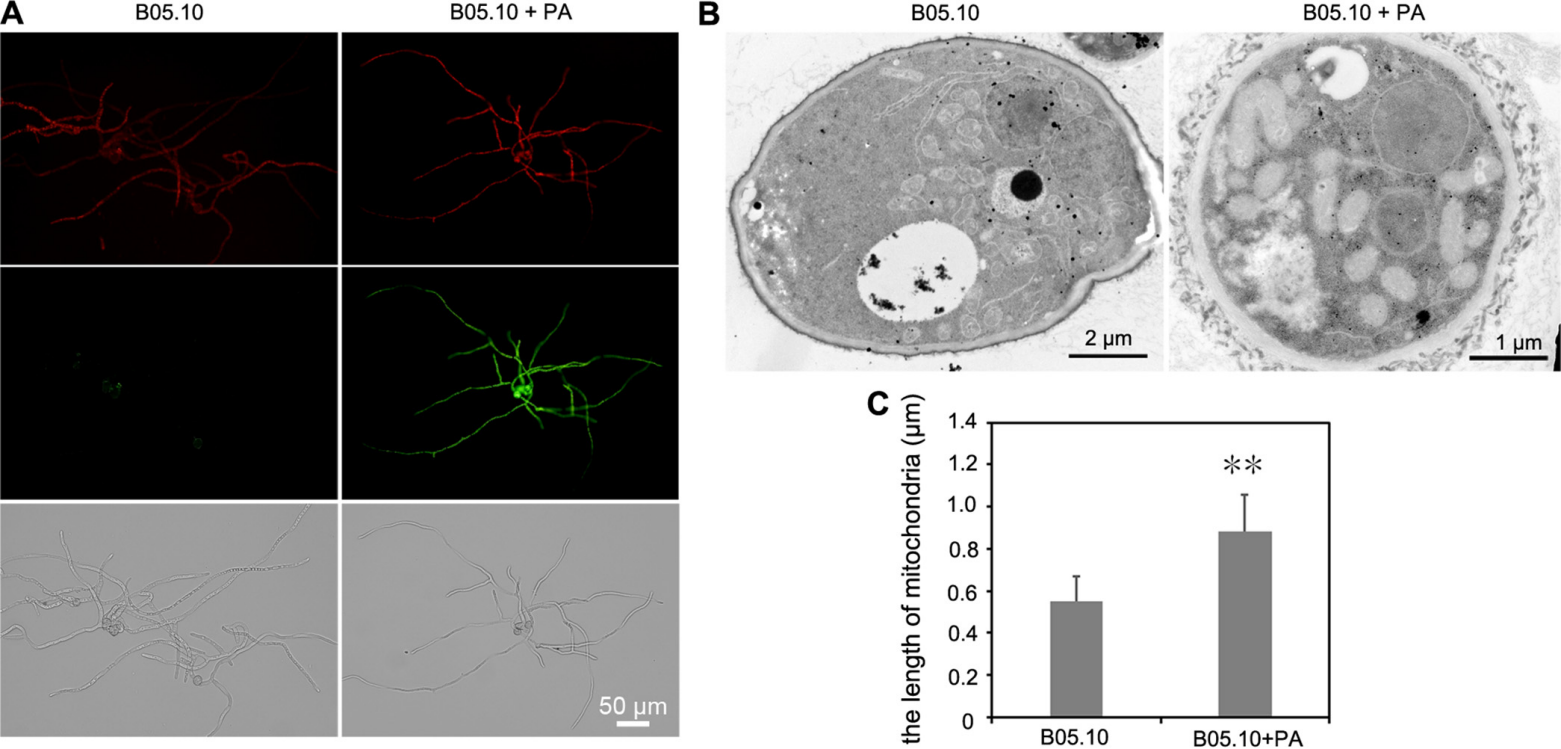
Effects of PA on Botrytis cinerea mitochondria. (A) Mitochondrial membrane potential on PA-treated mycelia after JC-1 straining. (B) Swollen mitochondria with markedly increased size were observed in *B. cinerea* treated with PA. (C) Diameters of mitochondria were measured. Error bars represent the standard deviations of three replicates, and asterisks represent statistically significant differences (*P* < 0.01).

Mitochondrial membrane potential (MtΔΨ) is a key indicator of cellular viability. A decrease in mitochondrial membrane potential is believed to be a point of no return in the apoptosis pathway. Mitochondrial dysfunction induced by PA treatment was investigated by MtΔΨ examination using the fluorescent dye JC-1. Exposure to PA resulted in an increase in the green fluorescence intensity, indicating a compromised MtΔΨ. In contrast, no green fluorescence was observed in the H_2_O-treated mycelia (Fig. 4A). The mitochondrial morphologies were examined using transmission electron microscopy (TEM). Mitochondrial swelling was observed in the PA-treated mycelia but not in the H_2_O-treated mycelia (Fig. 4B and C).

### Impact of PA on plasma membrane integrity.

**FIG 5:**
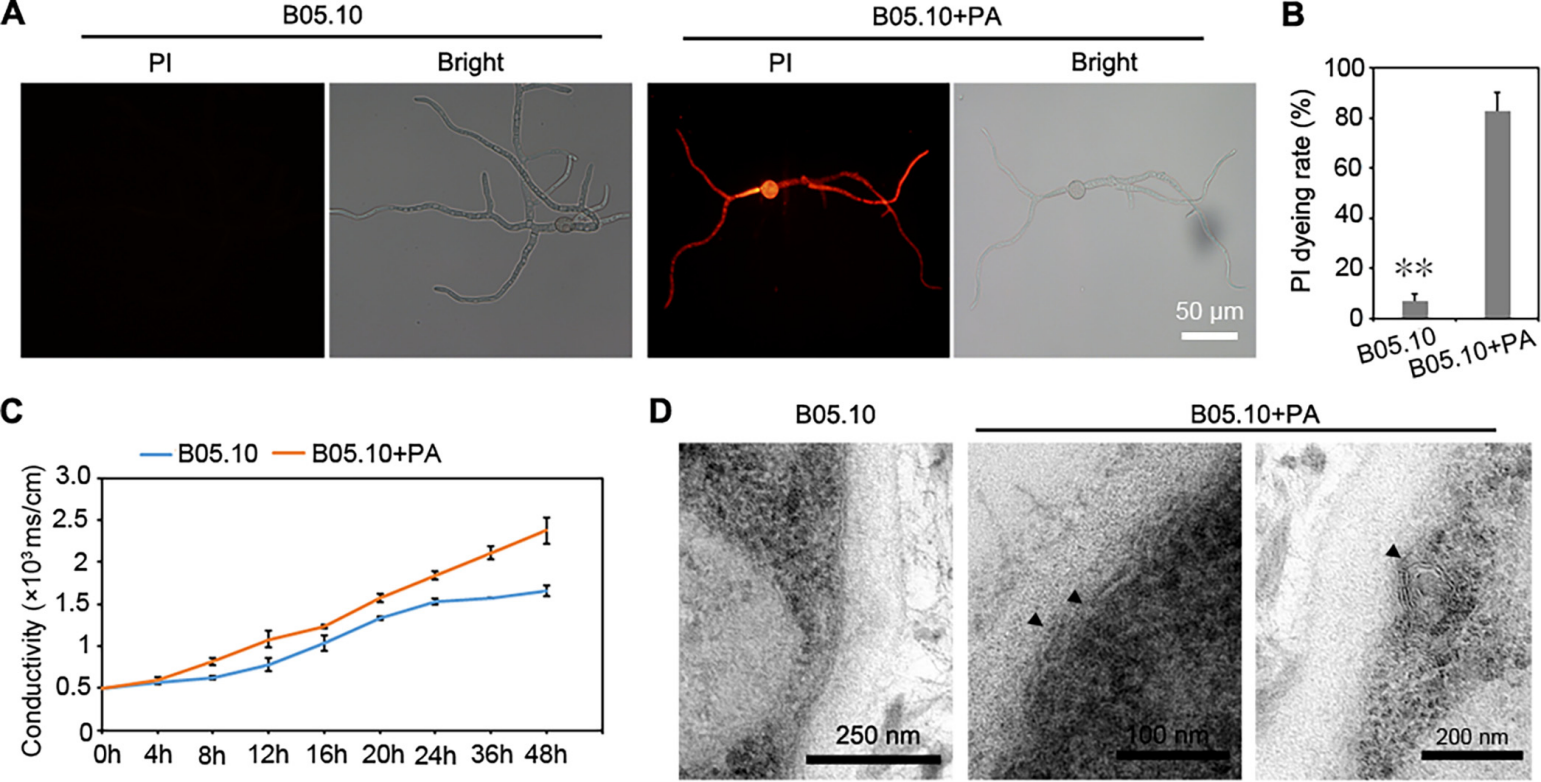
Effects of PA on the plasma membrane of Botrytis cinerea. (A) PI staining of mycelia after PA treatment. (B) Calculated fluorescence intensity on PA-treated mycelia after PI staining. (C) Conductivity analysis of mycelia after PA treatment. (D) PA-treated mycelia observed by TEM. Black arrowheads indicate the damaged plasma membrane.

Previous studies as well as the current study indicated that PA caused the exposure of phosphatidylserine on the surface of the plasma membrane. To obtain further information on this process, plasma membrane integrity was investigated by monitoring propidium iodide (PI) uptake, a process that requires significant damage on the cell membrane. Based on the fluorescence intensity, PA-treated mycelia were found to be extensively stained by PI (more than 90% of mycelia), while the H_2_O-treated mycelia displayed a polarized membrane that remained unstained (Fig. 5A and B). To further confirm the effect of PA on the plasma membrane, electric conductivity was measured. At 260 nm, a significant difference in electric conductivity was found between the PA-treated mycelia and the H_2_O-treated mycelia after 8 h. The electric conductivity of the PA-treated mycelia was significantly increased compared to that of the H_2_O-treated mycelia, along with time increase (Fig. 5C).

To further investigate the effect of PA on plasma membrane integrity of *B. cinerea*, TEM observation was carried out. Y shape gap and helix were found on the plasma membrane of PA-treated mycelia. In contrast, the plasma membrane of H_2_O-treated mycelia was smooth and complete (Fig. 5D). These results indicated that the plasma membrane integrity was destroyed by PA treatment and as a consequence the permeability of the membrane was increased.

### Ubiquitylation analysis of B05.10 treated with PA.

**FIG 6:**
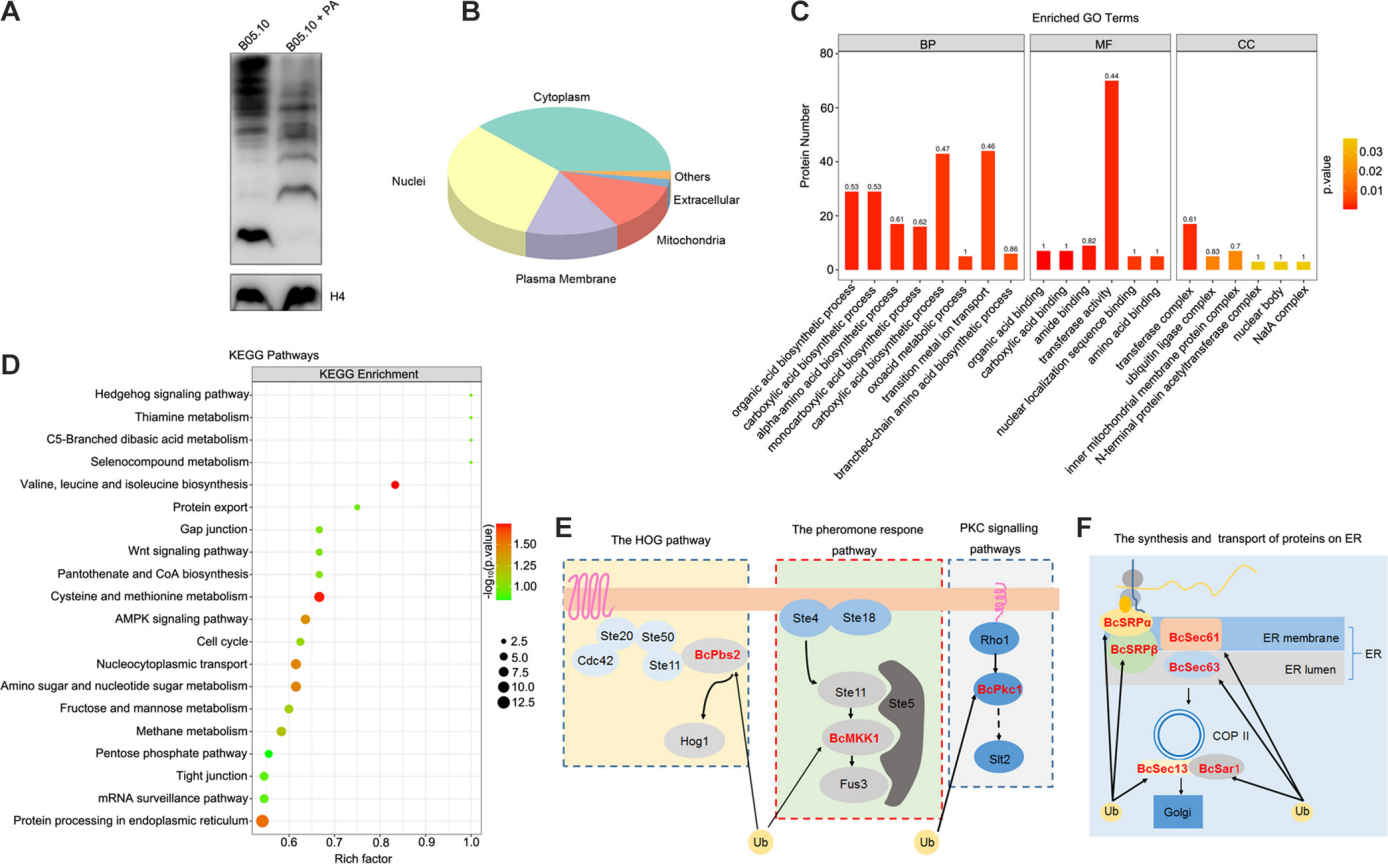
Ubiquitination in Botrytis cinerea treated with PA. (A) PA promoted ubiquitination in *B. cinerea*. (B) Ubiquitinated proteins. (C) Enrichment analysis of GO annotations in identified ubiquitinated proteins. (D) Enrichment analysis of KEGG pathways in identified ubiquitinated proteins. (E) Ubiquitinated proteins in signal pathways. (F) Synthesis and transport of ubiquitinated proteins on ER.

Previous studies have shown that ubiquitin is a key modulator of apoptosis. To explore the effect of PA on protein degradation, the ubiquitin levels in PA-treated and H_2_O-treated mycelia were measured. The results showed that PA promoted ubiquitin levels of total proteins in the PA-treated mycelia compared to those in the H_2_O-treated mycelia (Fig. 6A). To further investigate the ubiquitylated proteins induced by PA treatment, ubiquitinomics of the PA-treated and H_2_O-treated mycelia were assayed and analyzed. A total of 3,783 lysine ubiquitinated peptides were detected from 1,212 unique proteins. Ubiquitinated proteins mainly localized in cytoplasm, nuclei, plasma membrane, and mitochondria (Fig. 6B). To better understand the biological functions of ubiquitination, the identified ubiquitinated proteins were annotated by gene ontology (GO) enrichment analysis. The results showed that ubiquitinated proteins were involved in many biosynthetic processes, including the carboxylic acid biosynthetic process, transition metal ion transport, and transferase activity (Fig. 6C). To better understand which pathways ubiquitination regulated, an enrichment analysis of Kyoto Encyclopedia of Genes and Genomes (KEGG) pathways in the ubiquitinome was carried out. The results showed that the most significantly enriched KEGG pathways were related to amino acid biosynthesis, protein export, and gap junction (Fig. 6D).

Interestingly, three ubiquitinated proteins (BcPbs2, BcMkk1, and BcPkc1) involved in three signaling pathways were found. These three pathways are the high osmolarity glycerol (HOG) pathway, pheromone response pathway, and protein kinase C (PKC) signaling pathway, and they regulated the downstream signaling pathways after ubiquitination (Fig. 6E). In addition, six ubiquitinated proteins, BcSrpα, BcSrpβ, BcSec61, BcSec63, BcSec13, and BcSar1, were found to be involved in the synthesis and transport of proteins on the ER, including the signal recognition particle (Fig. 6F). BcMic60 (mitochondrial protein) was ubiquitinated (Table 1), which is very important for the normal mitochondrial crista structure (18). It was also found that extracellular acidosis increased the ubiquitination levels of Mic60, which decreased the Mic60 protein levels and induced mitochondrial injury (19).

**TABLE 1:** Mitochondrion-related proteins identified in ubiquitinome

Protein	Modified peptide	Description	The possibility of modifying sites (A score)	Post-translational modification location	Motif sequence
BCIN_01g10340	AMAEENEYDSEPNDEGEIEK(+114.04)RPGK	Bccyt1, putative cytochrome mitochondrial precursor protein (Botrytis cinerea BcDW1)	K20:Ubiquitin(K):0.00	K155	DEGEIEKRPGKLS
BCIN_01g07830	EAELEK(+114.04)GWKPK	Bcpam16, putative mitochondrial import inner membrane translocase subunit tim-16 protein (Botrytis cinerea BcDW1)	K6:Ubiquitin(K):56.71	K129	REAELEKGWKPKV
BCIN_04g02200	EITASDPEALQGQDDWATK(+114.04)R	Bcilv6, similar to mitochondrial acetolactate synthase small subunit (Botrytis cinerea T4)		K208	QDDWATKRLAELK
BCIN_04g03330	EK(+114.04)INLGVGAYR	BcPIO13, similar to mitochondrial aspartate aminotransferase (Botrytis cinerea T4)		K51	ADSFKEKINLGVG
BCIN_01g05650	GETIVTEK(+114.04)SITR	Bcmgm101, putative mitochondrial genome maintenance protein mgm101 (Botrytis cinerea BcDW1)		K180	ETIVTEKSITREY
BCIN_04g04500	GLKAEILGSFAPTTQKK(+114.04)	Similar to mitochondrial porin (voltage-dependent anion channel) (Botrytis cinerea T4)	K17:Ubiquitin(K):0.00	K148	APTTQKKGAKVNL
BCIN_05g04420	VIGAISEK(+114.04)DVM(+15.99)SFAQR	Bcmas1, putative mitochondrial processing peptidase beta subunit protein (Botrytis cinerea BcDW1)		K439	IGAISEKDVMSFA
BCIN_09g02420	AK(+114.04)VILEAK	Hypothetical protein BCIN_09g02420 (Botrytis cinerea B05.10)	K2:Ubiquitin(K):74.55	K253	VAESKAKVILEAK
BCIN_01g01720	LGK(+114.04)LSELTNTVNDLEK	Bcmic60, mitochondrial inner membrane protein (Botrytis cinerea B05.10)	K3:Ubiquitin(K):62.79	K463	REGRLGKLSELTN
BCIN_01g01830	NQFGEPNC(+57.02)GFQVK(+114.04)	Bcmix17 (Botrytis cinerea B05.10)	C8:Carbamidomethylation:1000.00	K129	NCGFQVKSFTNCM

**FIG 7:**
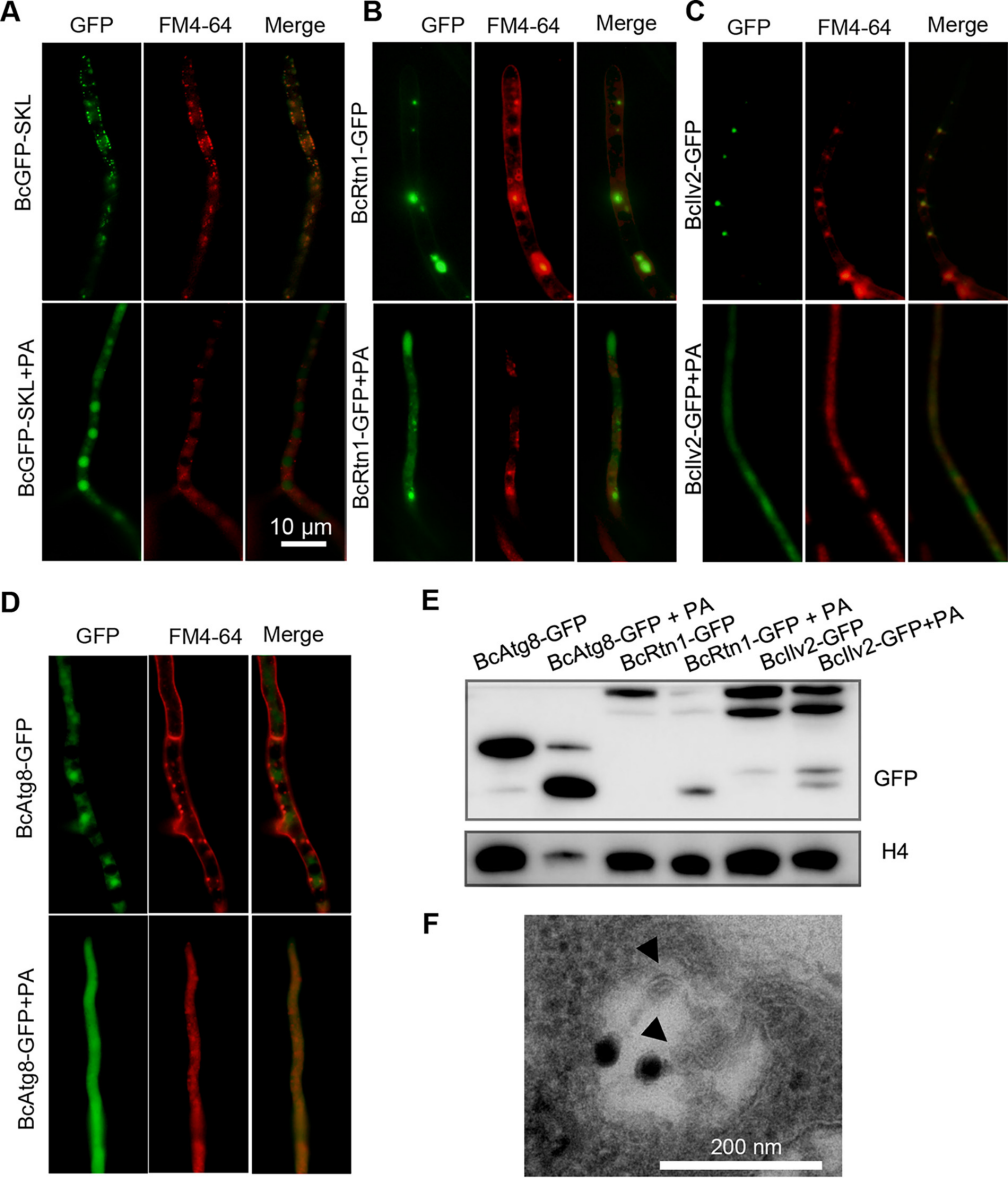
The promoting effect of PA on Botrytis cinerea autophagy. (A to D) Strains BcGFP-SKL (A), BcRtn1-GFP (B), BcIlv2-GFP (C), and GFP-BcAtg8 (D) were incubated in YEPD for 12 h and treated with PA for 4 h. Then fluorescence was observed with a microscope after staining with FM4-64 for 30 to 45 min. (E) Immunoblot analysis of GFP-BcAtg8 proteolysis. The upper bands show the intact BcRtn1-GFP, BcIlv2-GFP, and GFP-BcAtg8 and the lower free GFP. (F) Ultrastructural observation of induced autophagic vacuoles by TEM. Arrowheads indicate autophagosomes.

Two major pathways of protein degradation were found in eukaryotic cells, the ubiquitin proteasome pathway and the selective autophagy pathway (20, 21). Previous studies showed that many ubiquitinated proteins localized in peroxisome, mitochondrion, and ER. Whether PA participates in the induction of selective autophagy is unclear. To investigate whether PA induces pexophagy in *B. cinerea*, an alternative peroxisome marker, GFP-Ser-Lys-Leu (SKL), was used to indicate peroxisomes and to construct the BcGFP-SKL strain. Free green fluorescent protein (GFP) fluorescence was detected in the cytoplasm of the BcGFP-SKL strain without PA treatment, whereas free GFP fluorescence could be detected from the vacuole in the BcGFP-SKL strain treated with PA (Fig. 7A). The ER-phagy in *B. cinerea* was also investigated using BcRtn1 as the ER-phagy marker, which marks ER tubules, the edges of ER sheets, and the nuclear ER in a small fraction of cells (22). GFP fluorescence was detected in the cytoplasm and the vacuole in the BcRtn1-GFP strain with PA treatment, whereas GFP fluorescence in the BcRtn1-GFP strain without PA treatment mainly localized in the ER (Fig. 7B). The *BcIlv2* gene encodes an acetolactate synthase localized to the mitochondria (23). GFP fluorescence was observed in the cytoplasm and the vacuole in the BcIlv2-GFP strain with PA treatment, whereas the GFP fluorescence in B05.10 was mainly localized in mitochondria (Fig. 7C). GFP-BcAtg8 was introduced into the B05.10 strain for autophagic flux analysis. Without PA treatment, the GFP-BcAtg8 strain showed some cytoplasmic GFP, but not vacuolar GFP, whereas after PA treatment, the GFP-BcAtg8 strain showed GFP fluorescence in both vacuoles and cytoplasm (Fig. 7D). Next, the autophagic flux was monitored via immunoblotting. The proportion of free GFP in the PA-treated mycelia was significantly higher than that of the H_2_O-treated mycelia (Fig. 7E). Significantly more autophagosomes was observed in the vacuole after treatment with PA (Fig. 7F). These results further demonstrated that PA could induce autophagic activities.

### Transcriptome analysis of B05.10 treated with PA.

**FIG 8:**
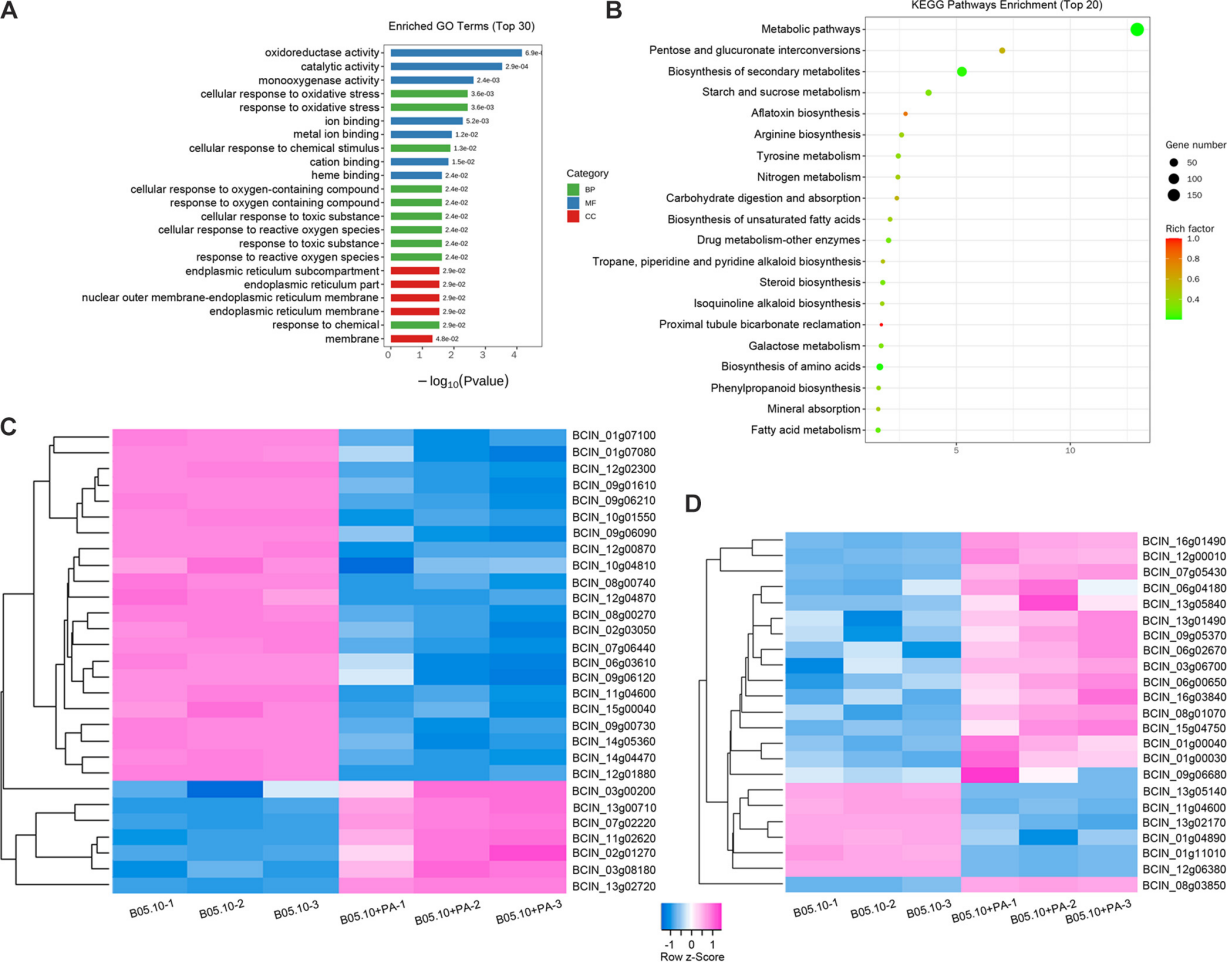
GO and KEGG enrichment analyses. (A) GO classification analysis of DEGs in PA-treated and H_2_O-treated mycelia of the B05.10 strain. (B) KEGG pathway analysis showing numbers of DEGs in PA-treated versus H_2_O-treated mycelia. (C and D) Heat maps showing relative expression for selected DEGs, multidrug transporter genes (C) and monooxygenase genes (D).

To further understand the mechanism of the antifungal action of PA, transcriptomes of the B05.10 mycelia exposed to PA or H_2_O were sequenced. In total, 1,012 differentially expressed genes (DEGs) were identified (log_2_ fold change >2 and adjusted *P* value < 0.05): 686 were upregulated and 326 were downregulated by PA treatment. To predict the functions of these DEGs, GO and KEGG enrichment analyses were performed. GO analysis grouped the 1,012 DEGs into three categories: those involved in biological processes, cellular components, and molecular functions. These DEGs were predicted to be primarily associated with response to oxidative stress, toxic substance, and oxygen species (Fig. 8A). The molecular functions of these DEGs were predicted to be involved in catalytic activities and binding. Interestingly, DEGs in the cellular component consisted mainly of ER-related genes. KEGG enrichment analysis of these DEGs indicated that these DEGs were involved in biological pathways related to metabolism and biosynthesis (Fig. 8B).

An interesting finding was that most genes related to multidrug transporters of the major facilitator superfamily (MFS), such as BcMfs1, BcHex4, and BcHex5, were downregulated by PA treatment, suggesting that PA could diminish the multidrug resistance of the fungus (Fig. 8C). Many monooxygenase genes for disintoxication, such as those encoding BcAba1, BcBoa3, and BcBoa4, were upregulated by PA treatment (Fig. 8D), which was supposed to help to increase the fungal resistance to exogenous substance (24).

### PA induces apoptosis independent of metacaspases.

**FIG 9:**
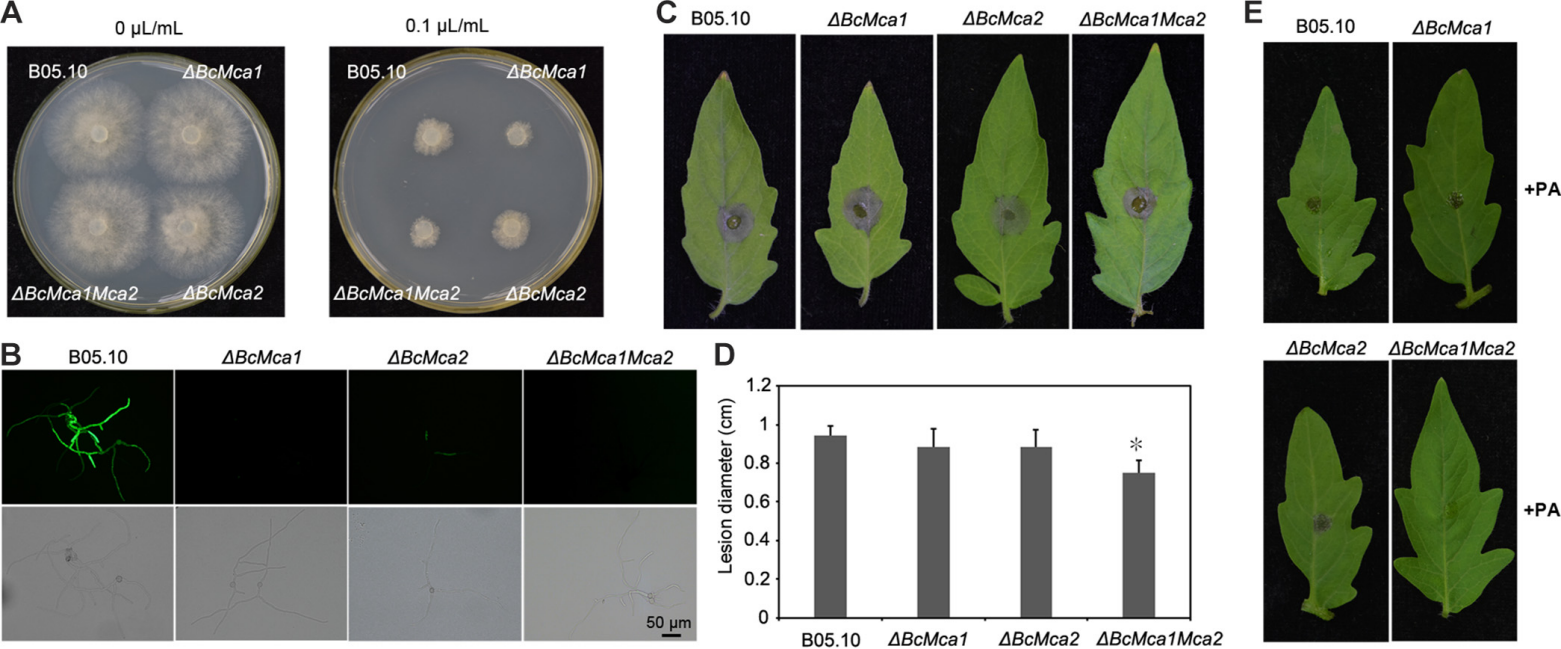
Functional analysis of metacaspases in Botrytis cinerea. (A) Δ*BcMca1*, Δ*BcMca2*, and Δ*BcMca1Mca2* strains showing no resistance on PDA medium after 3 days under PA stress. (B) ROS accumulation in the Δ*BcMca1*, Δ*BcMca2*, and Δ*BcMca1Mca2* strains was observed. (C) Δ*BcMca1* and Δ*BcMca2* strains did not affect the pathogenicity on tomato leaves, and Δ*BcMca1Mca2* strain was reduced in pathogenicity. (D) Lesion diameters were measured. Error bars represent the standard deviations for three replicates, and asterisks represent statistically significant differences (*P* < 0.01). (E) Detached tomato leaves inoculated with a *B. cinerea* conidial suspension and treated with PA at 0.05 μL/mL.

Previously studies in yeast indicated that the metacaspase Yca1 induced programmed cell death in response to oxidative stress (25). In A. flavus, PA could induce apoptosis that was mediated by intracellular metacaspases (10). To explore the function of metacaspases on PA-induced apoptosis in *B. cinerea*, two metacaspase genes, *BcMca1* and *BcMca2*, were studied. The expression levels of *BcMca1* and *BcMca2* in PA-treated and H_2_O-treated B05.10 mycelia were similar (Fig. S2), suggesting that the PA-induced apoptosis was independent of the metacaspase. To further confirm this conclusion, Δ*BcMca1* and Δ*BcMca2* single-gene deletion strains and a Δ*BcMca1Mca2* double-deletion strain were generated. To measure the sensitivity of the mutants to PA, the B05.10 strain and the Δ*BcMca1*, Δ*BcMca2*, and Δ*BcMca1Mca2* mutants were incubated on PDA plates supplemented with PA. After 4 days, the Δ*BcMca1* and Δ*BcMca1Mca2* mutants showed increased sensitivity to PA compared to that of B05.10. However, no difference was observed between Δ*BcMca2* and B05.10 (Fig. 9A). A previous study on *B. cinerea* indicated that PA could induce ROS accumulation, leading to the activation of programmed cell death. To investigate whether BcMca1 and BcMca2 are involved in apoptosis through ROS, intracellular ROS accumulation in the three mutants was examined. No ROS accumulation was observed in the three mutants compared to B05.10 (Fig. 9B). To further investigate the function of metacaspases in pathogenesis, pathogenicity assays were conducted for B05.10 and the three mutants. Only the Δ*BcMca1Mca2* mutant showed reduced pathogenicity compared to the single-gene mutants and B05.10 (Fig. 9C and D). The pathogenicities of B05.10 and the Δ*BcMca1*, Δ*BcMca2*, and Δ*BcMca1Mca2* mutants were further compared after treatment of the strains with PA. A pattern similar to that of the untreated strains was observed (Fig. 9E). These results indicated that the metacaspases play a nonessential role in stress-induced programmed cell death in *B. cinerea*.

## DISCUSSION

Gray mold caused by *B. cinerea* is a plant disease with substantial economic importance. Over the past several decades, fungicide-resistant strains of *B. cinerea* have emerged, even in the areas where no commercial fungicides had been used (26). Natural chemicals with antimicrobial properties are widely distributed in various forms of organisms. However, the molecular mechanisms underlying the antifungal effects of natural compounds on *B. cinerea* were barely understood, which has severely restricted the development of novel fungicides for this fungal species. Several previous studies reported the antifungal activity of PA on Aspergillus species, mainly by triggering metacaspase-dependent apoptosis (10, 27). In this study, the potential antifungal mechanism of PA on *B. cinerea* was investigated. Our results indicated that PA caused apoptosis in *B. cinerea*, which was dependent on ROS, autophagy, protein degradation, and plasma membrane integrity compromise but not metacaspase.

PA is abundant in the herb genus *Perilla* and can be used as an alternative substance to control fungal infection on fruits. Previous studies using quantitative structure–activity relationship (QSAR) analysis and transgenic rodent gene mutation (TGR) assays showed no evidence of mutagenic potential of PA (28–30). Therefore, PA is suitable to be used as a natural food preservative.

The ubiquitin proteasome system (UPS) is responsible for the regulated degradation of intracellular proteins and maintenance of protein turnover, which can either trigger or inhibit apoptosis (31, 32). The present study demonstrated that ubiquitination in PA-treated B05.10 mycelia was upregulated compared to that of H_2_O-treated mycelia. Three signal pathway-associated proteins were found to be ubiquitinated when B05.10 was exposed to PA. Ubiquitinated BcPbs1 was first found in *B. cinerea*, and its function is yet to be further elucidated. Ste7 is a mitogen-activated protein kinase kinase (MAPKK) that mediates pheromone signaling and the invasive growth pathways in Saccharomyces cerevisiae. Ubiquitinated Ste7 can specifically enhance the activation of Kss1 and lead to spurious activation of the invasive growth response (33, 34). The Pkc1-mediated signaling pathway that controls cell wall integrity can be negatively regulated by the deubiquitinating enzyme Ubp3 (35). Pkc1 is also involved in pexophagy but does not affect general autophagy (36). Although most studies on pexophagy in yeast have indicated an ubiquitin-independent process, ubiquitination has been demonstrated to be sufficient to induce pexophagy in mammalian cells. In the present study, BcPrx9 and BcPrx7 were found to be ubiquitinated during pexophagy. In ubiquitin-mediated autophagy, proteins or organelles are modified by ubiquitin and then recognized by the autophagy adaptors to initiate the formation of autophagosomes (21). ER-associated proteins are core components of protein translocation. In this study, six ER-associated proteins were found to be ubiquitinated as a result of PA treatment. These ubiquitination sites were first identified and need to be further elucidated in *B. cinerea*. In response to mitochondrial damage, mitochondrial proteins are ubiquitinated, which is essential for their sequestration and degradation within the lysosomes (37). Extracellular acidosis was found to damage the normal mitochondrial structure through promotion of Mic60 ubiquitin-dependent protein degradation (19). Consistent with this result, PA was found to be able to promote ubiquitination of mitochondrion-associated proteins, including BcMic60, BcPio13, and BcIlv6. In this study, mitophagy and ER-phagy were also promoted by PA treatment (BcIlv2-GFP and BcRtn1-GFP). We concluded that the autophagy was increased by ubiquitin through promotion of protein degradation.

PA inhibits mycelial growth and conidial germination in *B. cinerea*. Consistent with this result, PA was previously found to have antifungal properties against Aspergillus flavus, A. oryzae, A. niger, and Alternaria alternata on cherry tomatoes (38). Apoptosis is usually mediated by two key molecular signals: ROS and Ca^2+^ (39). After PA treatment, morphological characteristics reminiscent of apoptosis were observed in *B. cinerea*, which included intracellular Ca^2+^, ROS accumulation, MtΔΨ decrease, PS externalization, and DNA fragmentation. High levels of ROS in response to stress conditions can cause damage of proteins and cellular organelles. The ROS-generating organelles (such as mitochondria and peroxisome) can be removed by autophagy to restrict further ROS production (40). Free Ca^2+^ (efflux from the ER) ions with increased concentrations in cytosol can be taken up by mitochondria to trigger autophagy, presumably through the activation of calmodulin-independent kinase kinase β (41). In short, we concluded that PA can induce autophagy through multiple pathways, including Ca^2+^ and ROS.

Previous studies indicated the PA can cause the release of cytochrome *c* from mitochondria to cytoplasm and thus activate metacaspases (10). However, these studies did not investigate the expression and function of metacaspase in pathogens treated with PA but simply regarded the release of cytochrome *c* as a result of PA treatment. In fungi, apoptotic pathways can be either metacaspase dependent or independent (42). A previous study indicated that apoptosis induced by dihydrosphingosine and phytosphingosine against Aspergillus nidulans is independent of metacaspase but requires mitochondria (43). Consistent with this result, deletion of the two metacaspase genes in *B. cinerea* did not decrease the fungal sensitivity to PA. Consequently, we proposed that apoptosis induced by PA is independent of the two metacaspases in *B. cinerea*. The molecular mechanism beneath our observation in *B. cinerea* may be similar to that of the evolutionarily conserved caspase-independent apoptosis observed in mammals and Dictyostelium discoideum (44, 45).

**FIG 10:**
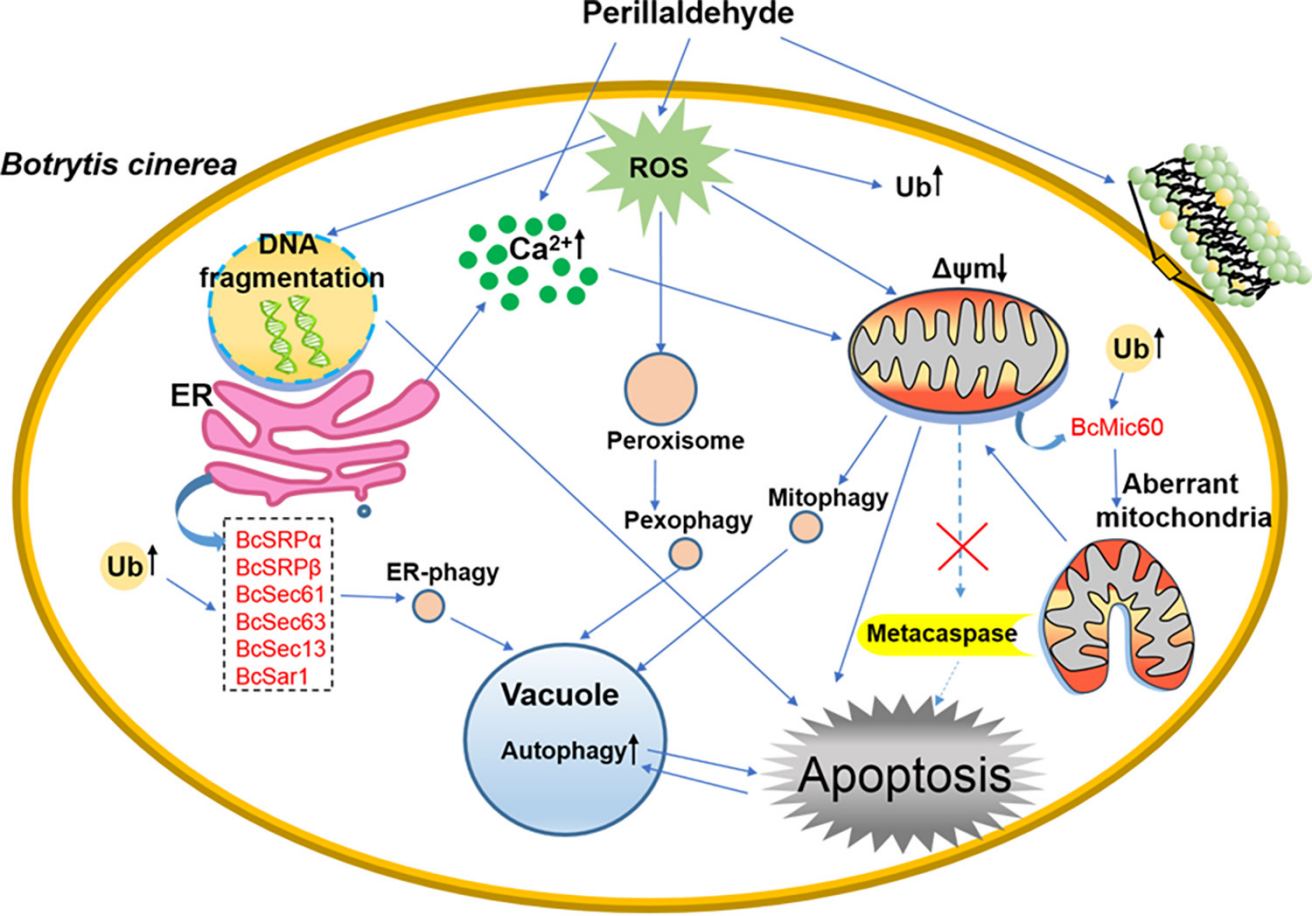
Model of PA-triggered apoptosis in Botrytis cinerea. PA directly disrupts plasma membrane integrity and induces intracellular ROS and Ca^2+^ accumulation, which causes depolarization of mitochondrial membrane potential. In addition, ROS accumulation can trigger DNA fragmentation. PA also promotes the ubiquitination; ultimately, excessive protein degradation causes autophagy. These results show that PA induced the apoptotic process via multiple pathways in *B. cinerea*, not a metacaspase-dependent pathway.

Based on the above results, we propose a mechanistic model to illustrate the function of PA on *B. cinerea* (Fig. 10). PA directly disrupts plasma membrane and induces an elevation of Ca^2+^ and ROS, which act as early signal mediators for apoptosis in *B. cinerea*. We speculate that ROS and Ca^2+^ may directly disrupt mitochondria, resulting in a significant depolarization of the MtΔψ. Furthermore, PA-induced accumulation of ROS can trigger DNA fragmentation and cause pexophagy. PA also promotes ubiquitination. Ultimately, excessive protein degradation causes autophagy that leads to cell death. PA induces apoptosis process via multiple pathways in *B. cinerea*, but not a metacaspase-dependent pathway.

## MATERIALS AND METHODS

### Chemicals and fungal strains.

Perillaldehyde (PA; CAS registry no. 18031-40-8) and BioTracker 640 red C2 (FM4-64) synaptic dye were purchased from Sigma-Aldrich (St. Louis, MO, USA). 2′,7′-Dichlorofluorescein diacetate (DCFH-DA), 4′,6-diamidino-2-phenylindole (DAPI), propidium iodide (PI), an annexin V-Fourier infrared transform chromatography (FITC) kit, a TUNEL kit, and Fluo-3/AM were purchased from Beyotime Co. Ltd. (Shanghai, China).

The *B. cinerea* wild-type strain B05.10 was maintained on potato dextrose agar (PDA) at 25°C as previously described (46). Fungal strains of F. oxysporum, *C. fructicola*, *N. clavispora*, R. solani, and *C. cassiicola*, as well as a strain of the oomycete *P. capsici*, were also maintained on PDA at 25°C. A strain of the bacterium R. solanacearum was maintained in LB broth at 30°C.

### *In vitro* testing of the inhibitory effect of PA on mycelial growth.

The fungal and oomycete strains were cultured on PDA medium containing PA at a concentration gradient of 0, 0.025, 0.05, 0.1, 0.2, and 0.4 μL/mL of medium. PDA plugs 5 mm in diameter were obtained from the edges of the colonies on 3-day-old cultures using a cork borer and transferred to freshly prepared PDA plates containing PA. The plates were incubated at 25°C in the dark. After 3 days, the diameter of the colonies was measured. For each strain, three plates were prepared for each PA concentration (as three biological repeats). In addition, conidia of B05.10 were incubated in potato dextrose broth (at 4.5 × 10^4^ conidia per mL) containing PA at the above-mentioned concentrations at 25°C, and the efficacy of PA on conidial germination was examined after 12 h.

### Testing the inhibitory effect of PA on pathogenesis.

Conidia of B05.10 were collected from 7-day-old PDA plates. The concentration of the conidia was measured by microscopic counting with a hemocytometer and adjusted to 4.5 × 10^4^ conidia per mL. Leaves detached from 4-week-old tomato plants were sprayed with PA at a concentration of 0, 0.025, 0.05, 0.1, 0.2 or 0.4 μL/mL, with 30 μL per leaf. The leaves were air dried for 4 h and then inoculated with the prepared conidial suspension at 20 μL per leaf. For the *in vivo* leaf test, PA at 0.4 μL/mL was sprayed on the leaves of tomato plants in pots (20 μL per leaf) and after 4 h, the leaves were sprayed with the conidial suspension at 20 μL per leaf. The inhibitory effect of PA on pathogenesis was also tested on detached fruits of tomato, grape, and strawberry. The fruits were obtained at commercial maturity and wounded at their equators. A 5-mm-diameter mycelial plug or 20 μL of conidial suspension was inoculated on the wounding site. The inoculated fruits were incubated in an airtight box, receiving one of two treatments: fumigation with PA or with H_2_O. After 3 days, the fruits were kept at 95% humidity at room temperature for another 3 days, and then the diameter of developing lesions on each fruit was measured.

### Fluorescence microscopy.

Conidia of B05.10 were incubated in yeast extract-peptone-dextrose (YEPD) liquid medium (4.5 × 10^4^ conidia per mL) at 25°C in a 120-rpm shaker. After 24 h, PA was added into the culture at a final concentration of 0.2 μL/mL. The culture was incubated under the same conditions for 4 h and then subjected to microscopic investigation. The cell viability and membrane integrity were analyzed by PI (20 mg/L), and the intracellular reactive oxygen species accumulation was analyzed by DCHF-DA (10 μM) staining. DNA and nuclear fragmentation were measured by TUNEL and DAPI assays. The intracellular Ca^2+^ levels under the PA treatment were analyzed using the nonfluorescent dye Fluo-3/AM. Phosphatidylserine (PS) exposure was detected by a FITC-coupled annexin V reaction with the annexin V-FITC kit. All the above-mentioned procedures followed the protocols provided by the corresponding manufacturers.

To examine whether PA affects autophagy, conidia of these strains (BcRtn1-GFP, BcIlv2-GFP, BcGFP-SKL, and GFP-BcAtg8) were cultured in YEPD liquid medium at 25°C for 24 h and then treated with PA for 4 h as described above. Mycelial samples were stained with FM 4-64, and the fluorescence was examined using an Olympus fluorescence microscope (Tokyo, Japan).

### TEM.

To examine autophagy and the plasma membrane, conidia of B05.10 were cultured in YEPD liquid medium at 25°C in a 120-rpm shaker for 24 h and then treated with PA for 4 h as described above. Transmission electron microscopy (TEM) was performed as previously described (46). Briefly, the mycelia were fixed in a fixative containing 2.5% (vol/vol) glutaraldehyde and 3% (vol/vol) paraformaldehyde, followed by postfixation in cacodylate buffer. After ethanol series dehydration, the sample was embedded in resin and sectioned. Sections were stained with uranyl acetate and lead citrate. Finally, the sample was examined and photographed under a JEM-1230 transmission electron microscope (JEOL, Tokyo, Japan).

### Transcriptome analysis.

The B05.10 strain was cultured in YEPD medium at 25°C for 24 h, then half was treated with PA at a final concentration of 0.2 μL/mL and the other half was treated with H_2_O at 25°C for 4 h. Total RNA was extracted from the treated mycelia using the TRIzol reagent (Magen, Guangzhou, China) by following the manufacturer’s protocol. The quality and concentration of the RNA samples were measured with the NanoDrop ND-2000 system (Thermo Scientific, USA). High-throughput transcriptome sequencing (RNA-seq) was performed with Illumina NovaSeq 6000 platform, from which 200-bp paired-end reads were obtained.

Differentially expressed genes (DEGs) between the PA-treated and H_2_O-treated B05.10 were identified using DESeq2. A log_2_ fold change of greater than 2 or less than −2 with a *P* value of <0.05 was considered differential expression. To explore the functions of the DEGs, gene ontology (GO) enrichment and Kyoto Encyclopedia of Genes and Genomes (KEGG) pathway analysis were performed using clusterProfiler R. The sequencing and sequence analysis were conducted by APTBIO Co., Ltd. (Shanghai, China).

### Ubiquitinome analysis.

B05.10 was cultured in YEPD medium at 25°C for 24 h, and then half was treated with PA at a final concentration of 0.2 μL/mL and the other half was treated with H_2_O at 25°C for 4 h. Samples of the treated mycelia were lysed in SDT (4% SDS, 100 mM Tris-HCl, 1 mM dithiothreitol [DTT] [pH 7.6]) buffer and from the lysate, total protein was extracted. Protein was quantified with the bicinchoninic acid (BCA) protein assay kit (Bio-Rad, Hercules, CA, USA). Protein was separated and then digested with trypsin. Lysine-ubiquitinated peptides were enriched using a PTMScan ubiquitin remnant motif (K-ε-GG) kit. Liquid chromatography-tandem mass spectrometry (LC-MS/MS) analysis was performed on a Q Exactive mass spectrometer (Thermo Fisher Scientific) that was coupled to an Easy nLC (Thermo Fisher Scientific) for 120 min. Sequences of the differentially expressed proteins were locally searched using the NCBI BLAST+ client software (ftp://ftp.ncbi.nlm.nih.gov/blast/executables/blast+/LATES), and then GO terms were obtained and sequences were annotated using the online program Blast2GO (https://www.blast2go.com). Classification of the annotated genes and pathways were performed using the KEGG (http://geneontology.org/).

### Obtaining knockout mutants and overexpression strain.

The two metacaspase genes, namely, *BcMca1* and *BcMca2*, were knocked out from the B05.10 strain by polyethylene glycol (PEG)-mediated protoplast transformation. Gene knockout vectors p*BcMca1* and p*BcMca2* were constructed using the fusion PCR method. Briefly, three DNA fragments were fused into one PCR product, which consisted of (from the 5′ to 3′ direction) a fragment at approximately 1 kb from upstream of the target gene, the open reading frame of the *HPH* gene, and a fragment approximately 1 kb from the downstream of the target gene. Protocols for protoplast preparation and PEG-mediated transformation of *B. cinerea* were described by Schumacher (47). The transformants were regenerated on selective medium (PDA containing 100 μg/mL of hygromycin). PCR were used to screen the putative gene deletion mutants to conform the presence of *HPH* and absence of the target genes. Single conidial isolates were obtained by spreading conidial suspensions on the selective medium and transferring single colonies to new plates.

GFP fusion constructs driven by the oliC promoter, namely, GFP-BcAtg8, BcRtn1-GFP, BcIlv2-GFP, and BcGFP-SKL, were generated according to the protocols described by Ren et al. (48). Nourseothricin-resistant transformants were obtained after transformation and screened by PCR, and GFP signal measurement was performed with an Olympus fluorescence microscope (Tokyo, Japan).

### Protein extraction and Western blotting.

The B05.10 strain and gene-overexpressing strains (GFPBcAtg8 strains, BcRtn1-GFP strains, and BcIlv2-GFP strains) were cultured in YEPD liquid medium at 25°C in a 120-rpm shaker for 24 h and then treated with PA for 4 h as described above. Mycelia were collected and resuspended in protein extraction buffer. An equal volume of the extracted protein from each strain was separated by SDS-PAGE gel and transferred to polyvinylidene fluoride membranes. The anti-GFP antibody 32146 was used at a 1:5,000 dilution for immunoblot analyses. The membranes were also detected with anti-histone 4 (Abcam, Cambridge, MA, USA) as a reference. The autophagy level was estimated by calculating the free GFP relative to the sum of intact BcRtn1-GFP/BcIlv2-GFP/GFP-BcAtg8 and free GFP.
