# Supplementary material for: Perillaldehyde Functions as a Potential Antifungal Agent by Triggering Metacaspase-Independent Apoptosis in Botrytis cinerea
Source: Microbiol Spectr. 2023 May 16;11(3):e00526-23. doi: 10.1128/spectrum.00526-23 (PMC10269628; doi:10.1128/spectrum.00526-23)
Supplement: Supplemental file 1 — Supplemental material. Download spectrum.00526-23-s0001.pdf, PDF file, 0.3 MB [file spectrum.00526-23-s0001.pdf]

SUPPLEMENTAL MATERIAL

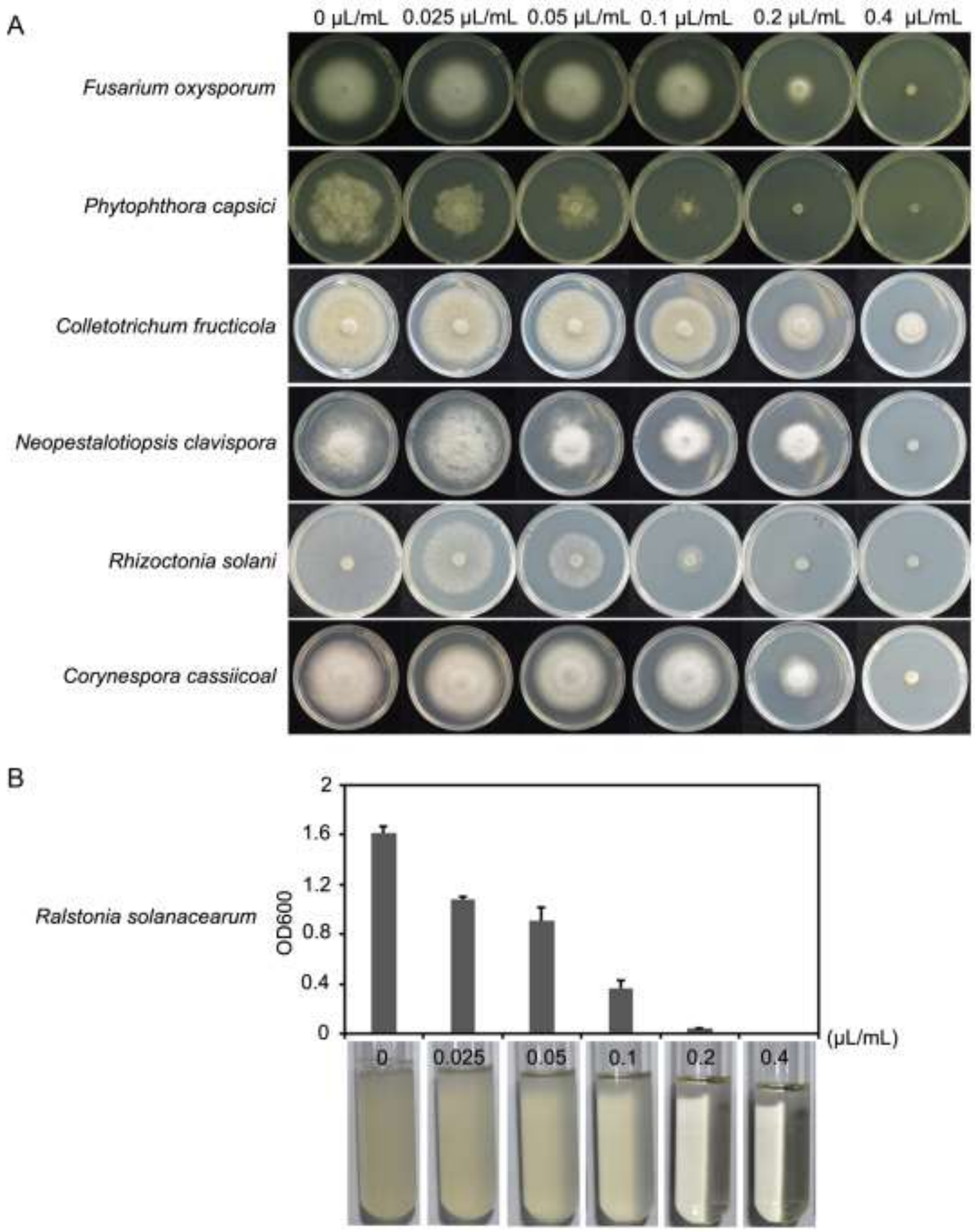

Fig S1 Antimicrobial activities of PA on the different fungus, oomycete, bacterium. (A) PA inhibited the mycelial expansion of *Fusarium oxysporum*, *Phytophthora capsici*, *Colletotrichum fructicola*, *Neopestalotiopsis clavispora*, *Rhizoctonia solani*, and

Corynespora cassiicola. (B) OD600 value measurement of PA against *Ralstonia solanacearum* with a concentration gradient.

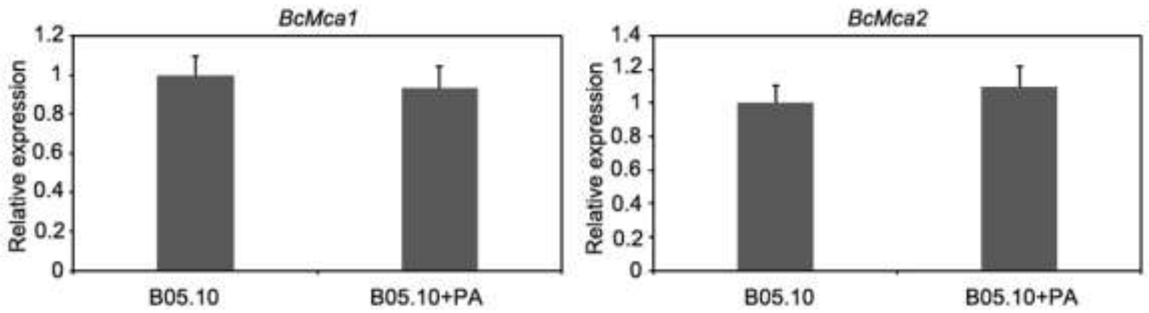

Fig S2 The expression levels of *BcMca1* and *BcMca2* determined via RT-qPCR.
